# Insights in opiates toxicity: impairment of human vascular mesenchymal stromal cells

**DOI:** 10.1007/s00414-023-02961-y

**Published:** 2023-02-14

**Authors:** Maria Carla Mazzotti, Gabriella Teti, Arianna Giorgetti, Francesco Carano, Guido Pelletti, Jennifer Paola Pascali, Mirella Falconi, Susi Pelotti, Paolo Fais

**Affiliations:** 1 Department of Medical and Surgical Sciences, Unit of Legal Medicine, University of Bologna, Via Irnerio 49, 40126 Bologna, Italy; 2 Department of Biomedical and Neuromotor Sciences, University of Bologna, via Irnerio 48, 40126 Bologna, Italy; 3 Department of Cardiologic, Thoracic and Vascular Sciences, University of Padova, Via Giustiniani, 2, 35127 Padua, Italy

**Keywords:** Opiate-related fatalities, Human vascular mesenchymal stromal cells, Morphine sulphate, In vitro, Forensic toxicology

## Abstract

The most common pulmonary findings in opiate-related fatalities are congestion and oedema, as well as acute and/or chronic alveolar haemorrhage, the cause of which is thought to be a damage to the capillary endothelium related to ischemia. Human vascular mesenchymal stromal cells (vMSCs) play a fundamental role in tissue regeneration and repair after endothelial cell injury, and they express opioid receptors. The aim of this study was to assess the effect of in vitro morphine exposure on the physiological activity and maintenance of human vMSCs. vMSCs were obtained from abdominal aorta fragments collected during surgery repair and were exposed to incremental doses (0.1 mM, 0.4 mM, 0.8 mM and 1 mM) of morphine sulphate for 7 days. The effect was investigated through cell viability assessment, proliferation assay, reactive oxygen species (ROS) detection assay, senescence-associated β-galactosidase assay, senescent-related markers (p21^WAF1/CIP1^ and p16^INK4^) and the apoptosis-related marker caspase 3. Moreover, an ultrastructural analysis by transmission electron microscopy and in vitro vascular differentiation were evaluated. Results showed a decrease of the cellular metabolic activity, a pro-oxidant and pro-senescence effect, an increase in intracellular ROS and the activation of the apoptosis signalling, as well as ultrastructural modifications and impairment of vascular differentiation after morphine treatment of vMSC. Although confirmation studies are required on real fatal opiate intoxications, the approach based on morphological and immunofluorescence methodologies may have a high potential also as a useful tool or as a complementary method in forensic pathology. The application of these techniques in the future may lead to the identification of new markers and morphological parameters useful as complementary investigations for drug-related deaths.

## Introduction

In the field of illicit drug use, heroin-related mortality is a complex phenomenon. There is a great variability in mortality rates attributable to heroin overdose, but most studies on longitudinal trends of overdose deaths, or overdose-related hospitalizations, show increases across time [1]. As other opiates, heroin and its metabolite morphine cause mental and gastrointestinal effects, as well as urinary retention; however, the most serious effect of opiates is the respiratory depression. The most common pulmonary findings in respiratory-related opiate fatalities are pulmonary oedema and congestion, as well as acute and/or chronic alveolar haemorrhage [2, 3]. Although this finding, called “heroin lung” or “noncardiogenic pulmonary edema” [4], is well-described, its pathogenesis is still obscure and several mechanisms have been proposed, including centrally induced respiratory depression, acute anaphylactic reaction, damage to the capillary endothelium related to ischemia (due to hypoventilation and hypoperfusion of the pulmonary vascular bed) and/or a direct toxic effect of the opiates on vascular endothelium [2, 5]. Several recent studies have shown that morphine might increase the vascular permeability and induce a dysfunction of the endothelium [6] and also the apoptosis of human endothelial cells via reactive oxygen species (ROS) pathways [7].

Tissue regeneration and repair are essential after endothelial cell injury and human vascular mesenchymal stromal cells (vMSCs) are heavily involved in this issue. vMSCs play a fundamental role in tissue repair and regeneration, being a versatile class of multipotent adult stem cells capable of self-renewal and provided with trophic property in secreting a wide range of growth factors, including vascular endothelial growth factor (VEGF). They also have immunomodulatory, anti-apoptotic and anti-inflammatory properties exerted in response to a mechanical damage or phlogosis. Opioid receptors are expressed on vMSCs and acute morphine exposition seems related to their functional impairment [8]; nonetheless, current knowledge on this topic remains limited [9].

The aim of this study was to assess the effect of in vitro opiate exposure on the physiological activity and maintenance of human vMSCs.

## Material and methods

### Sample collection and vascular mesenchymal stromal cell isolation and characterization

Abdominal aorta fragments were collected during surgery repair following the Code of Ethics of the World Medical Association from patients with abdominal aorta aneurism.

Explants were washed several times in phosphate-buffered saline (PBS), dissected in small pieces and cultured in minimal essential medium (MEM) (Gibco, Thermo Scientific, Monza, Italy) supplemented with 10% fetal bovine serum (FBS) (Gibco, Thermo Scientific, Monza, Italy), 10,000 U/ml penicillin–streptomycin at 37 °C and 5% CO_2_. After 2 weeks, the cells obtained were transferred to T25 cell culture flasks and subcultured for the following experiments in the same growing conditions as previously described.

The vMSCs obtained have been previously characterized for the expression of mesenchymal markers such as CD90 and CD105, in agreement with the scientific literature [10].

### Morphine sulphate treatment

vMSCs were exposed to 0.1 mM, 0.4 mM, 0.8 mM and 1 mM morphine sulphate (Molteni Farmaceutici S.p.A, Florence, Italy) diluted in MEM supplemented with 2% FBS for 7 days at 37 °C and 5% CO_2_. Control samples consisted in vMSCs grown in MEM supplemented with 2% FBS and MEM supplemented in 10% FBS for 7 days, with no exposure to morphine sulphate.

### Cell viability assessment

Tetrazolium bromide (MTT) assay was used to assess cell viability. vMSCs were seeded in triplicate (one triplicate for each morphine concentration) into a 96-well culture plate at the density of 8 × 10^3^ cells/well for 24 h. Then, the medium was changed with a fresh one containing the morphine sulphate solutions to be tested.

After 7 days, 10 µl of 5 µg/ml of MTT (Sigma-Aldrich, St. Louis, MO, USA) was added in each well and incubated for 3 h at 37 °C in 5% CO2. Formazan crystals were dissolved by adding dimethylsulphoxide (DMSO) in isopropanol (1:1). Optical density was measured, using a spectrophotometer microplate reader (LT-4000 Microplate reader, Labtech L.t.d, Heatfield, UK) at 578 nm (reference wavelength 690 nm).

Results were expressed in percentage as relative viability compared to control samples.

### Cell proliferation assay

Bromodeoxyuridine (BrdU) cell proliferation assay was performed by using Cell Proliferation ELISA BrdU (Roche Diagnostics GmbH, Mannheim, DE) according to the manufacturer’s protocol. In brief, vMSCs were seeded in triplicate into a 96-well culture plate at the density of 8 × 10^3^ cells/well for 24 h. Then, the medium was changed with a fresh one containing the morphine sulphate solutions to be tested. At the end of the treatment, 10 μM BrdU labeling solution was added in each well for 24 h. Then, the samples were fixed and incubated with anti-BrdU antibody peroxidase conjugated, for 90 min at room temperature. Following three washes in PBS, tetramethyl–benzidine (TMB) substrate solution was added for 20 min at room temperature, and the reaction was stopped with 1 M H_2_SO_4_. The optical density was measured using a spectrophotometer microplate reader (LT-4000, LabTech, Euroclone, Milan, Italy) at a wavelength of 450 nm and a reference wavelength of 690 nm. Results were expressed in percentage as relative values compared to control vMSCs.

### Reactive oxygen species detection assay

Carboxy-H_2_DCFDA fluorescent dye (Invitrogen, Carlsbad, CA, USA) was employed to quantitate the oxidative stress induced by 7 days morphine exposition. vMSCs were seeded in triplicate into a 96-well culture plate at the density of 1 × 10^4^ cells/well for 24 h. Then, the medium was changed with a fresh one containing the morphine sulphate solutions to be tested. At the end of the treatment, samples were washed in PBS and 5 μM carboxy-H_2_DCFDA ROS detection probe (Thermo Fisher Scientific, Monza, Italy) diluted in MEM supplemented with 2% FCS was added for 3 h at 37 °C and 5% CO_2_.

As positive ROS control samples, vMSCs treated with 200 µM H_2_O_2_ in MEM supplemented with 2% FCS were added. A fluorimeter microplate reader (Glomax, Promega Corporation, Madison, WI, USA) with excitation and emission wavelengths set respectively at 492 nm and 517 nm was used to detect the fluorescent signal. Results were expressed as relative percentage compared to control samples.

### Senescence-associated β-galactosidase assay (SA-β-Gal)

Senescence was investigated by assessing SA-β-Gal biomarkers. To this scope, vMSCs were seeded in triplicate into a 96-well culture plate at the density of 1 × 104 cells/well for 24 h. Then, the medium was changed with a fresh one containing the morphine sulphate solutions. At the end of the treatment, samples were washed in PBS, fixed with 2% formaldehyde for 30 min and stained with the cell event senescent green probe (Invitrogen, Thermo Fisher Scientific, Eugene, OR, USA), a sensitive fluorescent substrate for the β-galactosidase enzyme, diluted 1:1000 in the staining buffer, for 1 h and 30 min at 37 °C, in a CO_2_ free environment. At the end of the incubation, samples were washed in PBS and the fluorescent signal was measured using a fluorescent microplate reader (GloMax Discover System, GM3000, Promega Corporation, Madison, WI, USA) at an excitation wavelength of 490 nm and an emission wavelength of 514 nm.

### Ultrastructural analysis by transmission electron microscopy (TEM)

vMSCs were seeded on cover glasses at the density of 1.5 × 10^4^ cells/glass for 24 h. Then, the medium was changed with a fresh one containing the morphine sulphate solutions to be tested. At the end of the treatment, samples were washed with PBS and fixed with 2.5% glutaraldehyde in 0.1 M phosphate buffer for 30 min at 4 °C, and they were post fixed with a solution of 1% OsO_4_ in 0.1 M phosphate buffer for 30 min at room temperature. After some washes in 0.15 M phosphate buffer, the samples were dehydrated in a graded series of acetone and embedded in Epon resin (Sigma-Aldrich, St. Louis, MO, USA). Ultrathin sections were counterstained with uranyl acetate and lead citrate and observed under a Philips CM100 (FEI Italia Srl, Milan, Italy). The images were digitally captured by an SIS Megaview III CCD camera (FEI Italia Srl, Milan, Italy). Any modification in nucleus and cellular organelles in exposed cells compared to controls was qualitatively assessed.

### Analysis of protein expression by western blot

RIPA-modified cell lysis buffer (Pierce, Thermo Fisher Scientific, Monza, Italy) supplemented with 25 μmol/l protease inhibitor cocktail (Pierce, Thermo Fisher Scientific, Monza, Italy) and 1 μl of β-mercapto-ethanol (Sigma-Aldrich, St. Louis, MO, USA) was used to extract total proteins from vMSCs at the end of each treatment. Following protein quantification by Bradford assay (Sigma-Aldrich, St. Louis, MO, USA), 15 µg of lysate was loaded and separated with 4–12% sodium dodecyl sulphate polyacrylamide gel electrophoresis (SDS-PAGE) and subsequently it was transferred onto a nitrocellulose membrane (GE Healthcare, Amersham, UK). Then, the membranes were incubated with 5% BSA (blocking reagent) to remove the nonspecific binding proteins, followed by incubation with the primary antibodies against rabbit anti-p21^WAF1/Cip1^ antibody (Cell Signaling Technologies, Euroclone, Milan, Italy), rabbit anti-p16^INK4A^ antibody (Cell Signaling Technologies, Euroclone, Milan, Italy), rabbit anti caspase3 antibody (Cell Signaling Technologies, Euroclone, Milan, Italy) and anti β-tubulin antibody (Cell Signaling Technologies, Euroclone, Milan, Italy), diluted 1:1000 in blocking reagent at 4 °C, overnight. After washes with TBS-tween buffer, samples were incubated with HRP-linked anti-rabbit antibody, diluted 1:2000 in TBS-tween buffer (Sigma-Aldrich, St Louis, MO, USA). The antibody signal was visualized by the enhancement chemiluminescence system (Pierce, Thermo Fisher Scientific, Monza, Italy). Images were obtained by using an IBright Western Blot Imaging System (Thermo Fisher Scientific, MA, USA). Finally, the densitometric analysis was performed using ImageJ software (National Institutes of Health, USA). The intensities of the specific protein bands were corrected for equal β-tubulin loading and they were expressed as relative value compared to the intensity of the control (CTR) sample. Data showed the median ± SD of three independent experiments.

### In vitro vascular differentiation

vMSCs were seeded on cover glasses at the density of 1.5 × 10^4^ cells/glass for 24 h. Then, the medium was changed with a fresh one containing 50 ng/ml of vascular endothelial growth factor (VEGF) for 7 days at 37° and 5% CO_2_, followed by morphine sulphate treatment for other 7 days at 37 °C and 5% CO_2_. At the end of the treatment, the samples were fixed in 4% paraformaldehyde in PBS for 30 min at 4 °C, followed by a permealization step in 0.1% Triton X-100 for 5 min at 4 °C. After some washes in PBS, the samples were covered with 2.5% bovine serum albumin (BSA) (Sigma-Aldrich, St. Louis, MO, USA) diluted in PBS (blocking reagent), for 30 min at room temperature (RT), followed by incubation in mouse anti-human CD31 antibody (Origene, Thermo Fisher Scientific, Monza, Italy) diluted 1:100 in blocking reagent, overnight at 4 °C. Then, the samples were washed in PBS and incubated for 1 h and 30 min at 37 °C with secondary antibody anti-mouse IgG–Cy3 conjugated (Sigma-Aldrich, St. Louis, MO, USA), diluted 1:2000 in PBS. Samples were rinsed with PBS, counterstained with DAPI and mounted in vectashield medium (Vector Laboratories, Inc., Burlingame, CA, USA). The fluorescence microscopy Eclipse E800 Nikon (Nikon, Tokyo, Japan) was utilized to observe the samples.

### Statistical analysis

Statistical analysis was carried out using Prism 5.0 software (GraphPad, San Diego, CA, USA) applying a one-way ANOVA followed by Tukey’s multiple comparison test. The differences were considered significant at *p* < 0.05.

## Results

### vMSC viability after morphine sulphate treatment

**Fig. 1:**
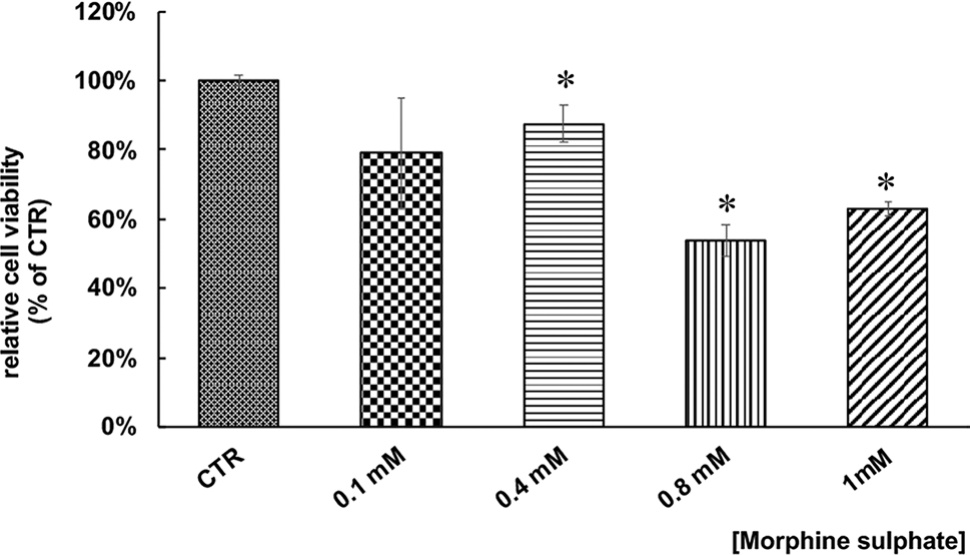
Effect of morphine sulphate on the viability of vMSC exposed to different concentrations of morphine (0–1 mM) for 7 days. At the end of the treatment, a MTT assay was carried out. Data is expressed in percentage as cell viability relative to control samples (CTR, 100%) and as mean ± standard error of the mean (SEM) of three independent experiments run in triplicate. Statistical significance was set as *p < 0.05, vs. CTR

In vitro cellular toxicity induced by different concentrations of morphine sulphate was investigated by MTT assay. Results showed a dose-dependent decrease of cell viability (Fig. 1) after 7 days of treatment. Particularly, samples exposed to 0.4, 0.8 and 1 mM morphine showed a statistically significant difference compared to control samples, reaching a reduction of 54% at 0.8 mM (*p* < 0.05) and 63% at 1 mM (*p* < 0.05). No statistically significant difference between samples exposed to 0.8 mM and 1 mM of morphine sulphate was seen.

### vMSC proliferation after morphine sulphate treatment

**Fig. 2:**
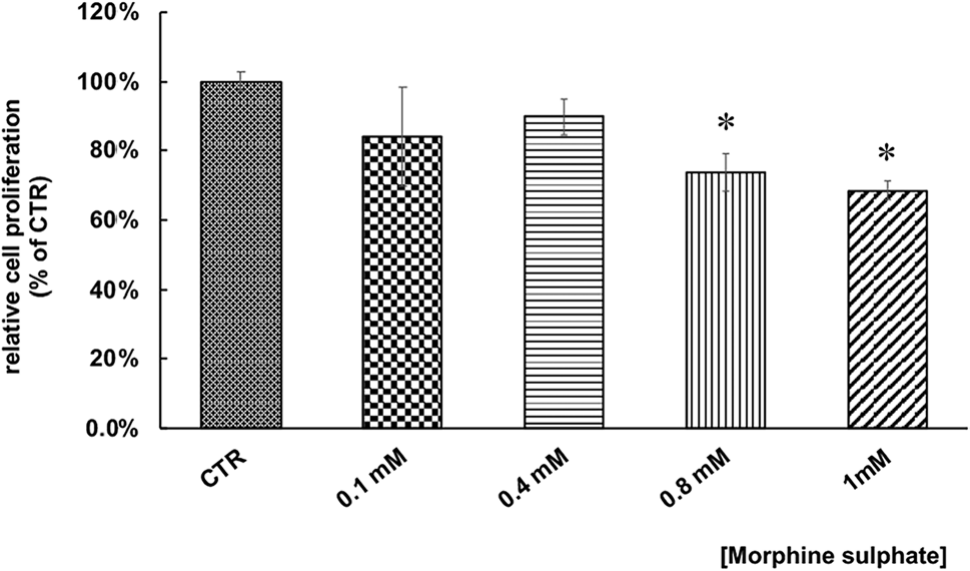
Effect of morphine sulphate on the proliferation of vMSC exposed to different concentration (0–1 M) for 7 days. At the end of the treatment, a BrdU assay was carried out. Data is expressed in percentage as cell proliferation relative to control samples (100%) and they are expressed as mean ± SEM of three independent experiments run in triplicate. Statistical significance was set as *p < 0.05, vs. control samples (CRT)

Based on cell viability results, in order to verify a potential reduction of cell proliferation induced by morphine treatment, a BrdU assay was carried out on vMSCs exposed to different concentration of morphine sulphate. Results showed a significant growth inhibition compared to control samples at the concentration of 0.8 mM (*p* < 0.05) and 1 mM (*p* < 0.05) of morphine sulphate (Fig. 2), but not at lower doses, suggesting an influence of the drug on cell cycle distribution, a pro-oxidant and pro-senescence effect [11].

### ROS generation in vMSCs after morphine sulphate exposition

**Fig. 3:**
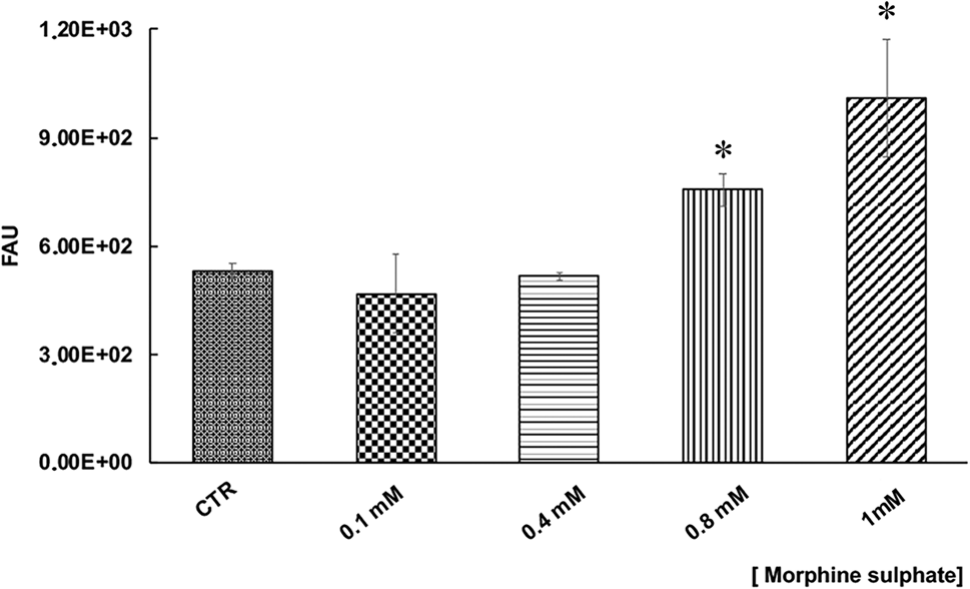
Effect of morphine sulphate on the total amount of cellular ROS in vMSCs exposed to different concentrations (0–1 mM) for 7 days. At the end of the treatment, a carboxy-H_2_DCFDA assay was carried out. Data is expressed in fluorescent arbitrary units (FAU) and they are expressed as mean ± SEM of three independent experiments run in triplicate. Statistical significance was set as *p < 0.05, vs. control samples (CRT)

In order to assess the effect of morphine sulphate on ROS generation, a carboxy-H2DCFDA assay was carried out on vMSCs exposed to different concentrations of the drug. A significant increase in intracellular ROS was observed in vMSCs exposed to 0.8 mM (*p* < 0.05) and 1 mM (*p* < 0.05) compared to control samples (Fig. 3), confirming a pro-oxidant effect of morphine sulphate.

### Expression of the apoptotic marker caspase 3

**Fig. 4:**
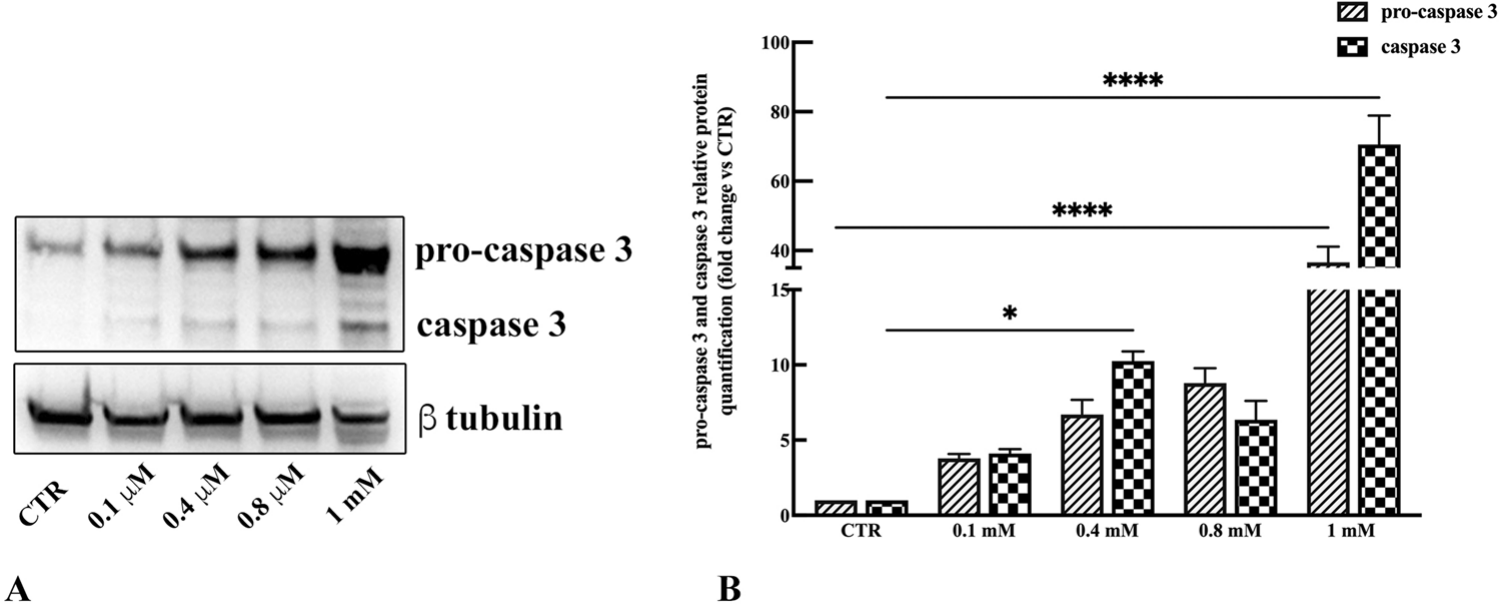
**A** Representative western blot images showing pro-caspase 3 and caspase 3 expression in vMSCs exposed to different concentrations of morphine sulphate. **B** Relative amounts of pro-caspase 3 and caspase 3 expression normalized to the intensity of β-tubulin and represented as fold increase relative to control cells (CTR). Western blot was performed in duplicate, and the relative quantification is expressed as mean value ± SD. **** represents a significant difference compared to CTR, *p* < 0.0001; * represents a significant difference compared to CTR, *p* < 0.05

The expression of caspase 3, a cysteine-aspartic acid protease which plays a key role in the execution phase of programmed cell death or apoptosis, was investigated in vMSCs exposed to morphine sulphate with the final aim to detect cell death induced by the treatment. Increase of pro-caspase 3 and especially cleavage of caspase 3 indicate an activation of the apoptotic signalling. Results showed an increase of the precursor pro-caspase 3 dose dependent (Fig. 4A) and a clear signal corresponding to the cleaved fragment of caspase 3 only in samples exposed to 1 mM of morphine sulphate (Fig. 4A). Densitometric analysis validates these data, demonstrating a 60-fold increase of the precursor protein pro-caspase 3 (*p* < 0.0001) and a 36-fold increase of the cleaved fragment caspase 3 (*p* < 0.0001) (Fig. 4B), indicating the activation of apoptosis.

### Evaluation of senescence biomarker on vMSCs exposed to morphine sulphate

**Fig. 5:**
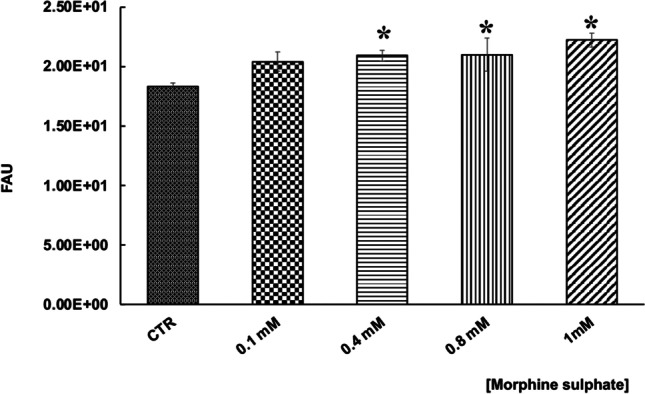
Effect of morphine sulphate on induction of cellular senescence in vMSCs exposed to different concentration (0–1 mM) for 7 days. At the end of the treatment, a SA-β-gal assay was carried out. Data are expressed in fluorescent arbitrary units (FAU) and they are expressed as mean ± SEM of three independent experiments run in triplicate. Statistical significance was set as *p < 0.05, vs. control samples (CRT)

SA-β-gal assay was then carried out to demonstrate a pro-senescent effect of morphine on vMSCs. A light but constant increase of the fluorescence signal was observed in all the samples compared to control ones (Fig. 5), suggesting a dose-dependent relation between cellular senescence and morphine concentration.

**Fig. 6:**
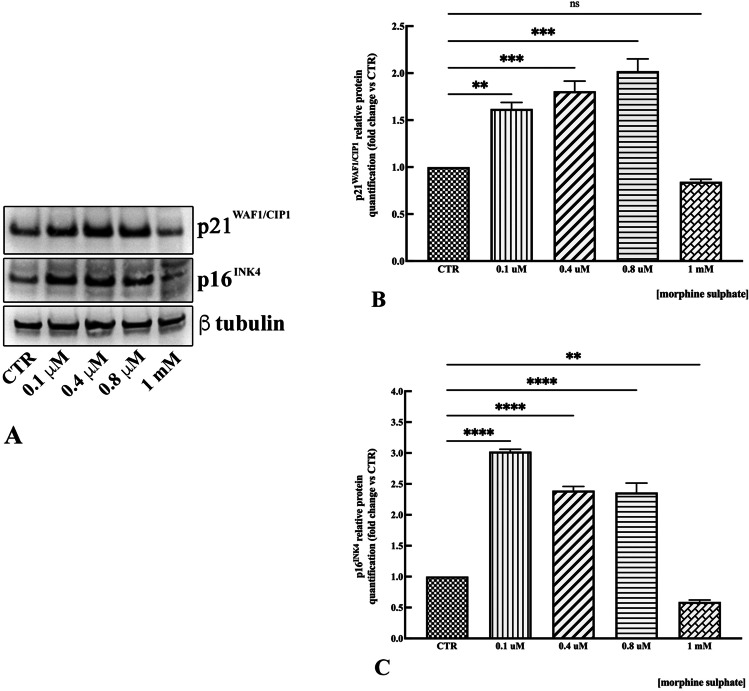
**A** Representative western blot images showing p21^WAF1/CIP1^ and p16^INK4^ expression in vMSCs exposed to different concentrations of morphine sulphate. **B** Relative amounts of p21^WAF1/CIP1^ expression normalized to the intensity of β-tubulin and represented as fold increase relative to control cells (CTR). Western blot was performed in duplicate, and the relative quantification is expressed as mean value ± SD. *** represents a significant difference compared to CTR, *p* < 0.001; ** represents a significant difference compared to CTR, *p* < 0.01; (ns) represents the lack of a significant difference compared to CTR. **C** Relative amounts of p16.^INK4^ expression normalized to the intensity of β-tubulin and represented as fold increase relative to control cells (CTR). Western blot was performed in duplicate, and the relative quantification is expressed as mean value ± SD. **** represents a significant difference compared to CTR, *p* < 0.0001; ** represents a significant difference compared to CTR, *p* < 0.01

In order to validate a senescent phenotype, the expression of the cyclin-dependent kinase inhibitors (CDKIs) p21^WAF1/CIP1^ and p16^INK4^ was investigated by western blot. Results showed an upregulation of the protein p21^WAF1/CIP1^ dose dependent, from 0.1 to 0.8 mM (Fig. 6A and B), followed by a downregulation of the protein at 1 mM of morphine sulphate treatment (Fig. 6A). Quantitative analysis showed a twofold highest increase of p21^WAF1/CIP1^ in samples treated with 0.8 mM (*p* < 0.001), while a downregulation of the protein to the level of control samples is observed at 1 mM of morphine sulphate treatment (Fig. 6B).

The expression of p16^INK4^ is threefold higher in cells exposed to 0.1 mM of morphine sulphate (*p* < 0.0001) (Fig. 6A and C), followed by a twofold increase of expression in samples treated with 0.4 and 0.8 mM (*p* < 0.0001) (Fig. 6A and C). A downregulation of 0.4 folds (*p* < 0.01) is observed in samples exposed to 1 mM of morphine sulphate (Fig. 6A and C).

### Ultrastructural analysis of vMSCs exposed to morphine sulphate

To better investigate the cellular behaviour toward the toxicity induced by morphine sulphate, an ultrastructural analysis by TEM was carried out. The aim was to detect any modification in nucleus and cellular organelles which could allow to elucidate the potential mechanisms underpinning cellular compensation to morphine sulphate treatment.

**Fig. 7:**
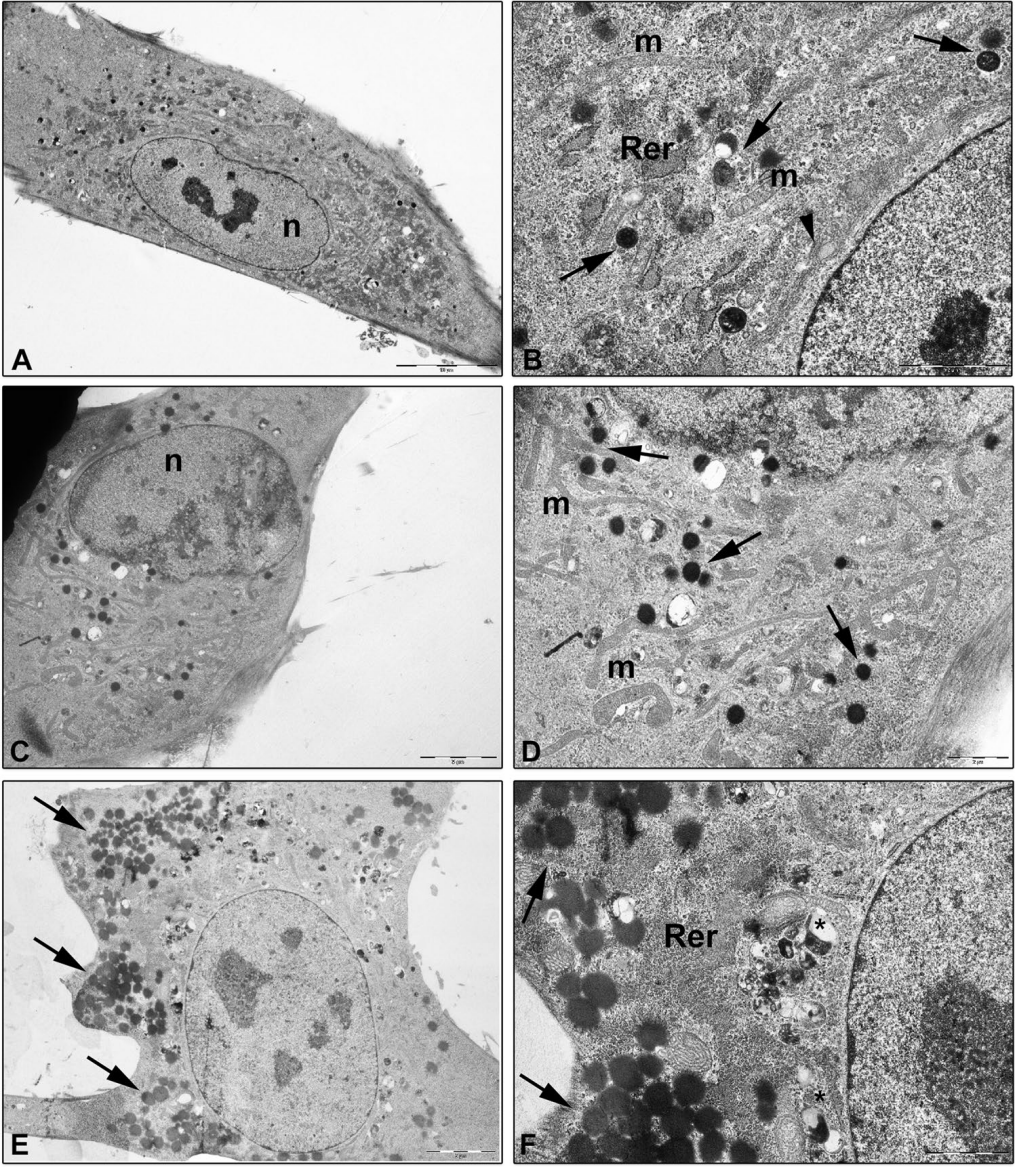
TEM ultrastructural analysis of vMSCs exposed to 0.8 and 1 mM of morphine sulphate for 7 days. **A** Control vMSCs. Cells showed a fibroblast-shape morphology with nucleus (n) and nucleoli well detected (bar: 10 µm). **B** Detail of vMSC cytoplasm showing mitochondria (m), primary lysosomes (arrow), rough endoplasmic reticulum (RER) and Golgi complex (arrowhead) (bar: 2 µm). **C** vMSCs after 0.8 mM of morphine sulphate treatment. Cells showed a round-shape morphology. Nucleus (n) and cytoplasm organelles are still detected (bar: 5 µm). **D** Detail of vMSC cytoplasm showing numerous long shape mitochondria (m) and several primary lysosomes (arrow) (bar: 2 µm). **E** vMSCs after 1 mM of morphine sulphate treatment. A proliferation of primary lysosome (arrow) scattered in the cytoplasm is observed (bar: 5 µm). **F** Primary lysosomes (arrow), secondary lysosomes, autophagic vesicles (*) and irregular RER are detected (bar: 2 µm)

Control cells demonstrated a fibroblast-shape morphology, with well-preserved nucleus and nucleoli and plasma membrane (Fig. 7A). In the cytoplasm, several cellular organelles such as mitochondria, rough endoplasmic reticulum (RER) and Golgi complex were easily detected. They showed normal size and cytoplasmic distribution, compatible with an active and healthy condition of the cell (Fig. 7B). Some lysosomes were also detected in the cytoplasm (Fig. 7B).

After 0.1 and 0.4 mM morphine exposure, the morphology of vMSCs was similar to controls.

After 0.8 mM morphine exposition, vMSCs showed a round-shape morphology (Fig. 7C). Several long-shaped mitochondria and primary lysosomes were detected in the cytoplasm (Fig. 7D).

After 1 mM of morphine exposition, a strong proliferation of primary lysosomes and autophagic vesicles was observed in the cytoplasm of the vMSCs (Fig. 7E).

### Vascular differentiation of vMSCs

In order to demonstrate a potential morphine sulphate–induced impairment of the ability of vMSCs to differentiate toward a vascular phenotype, the expression of the endothelial marker CD31 was investigated in cells exposed to VEGF and/or morphine sulphate for 7 days by immunofluorescence.

**Fig. 8:**
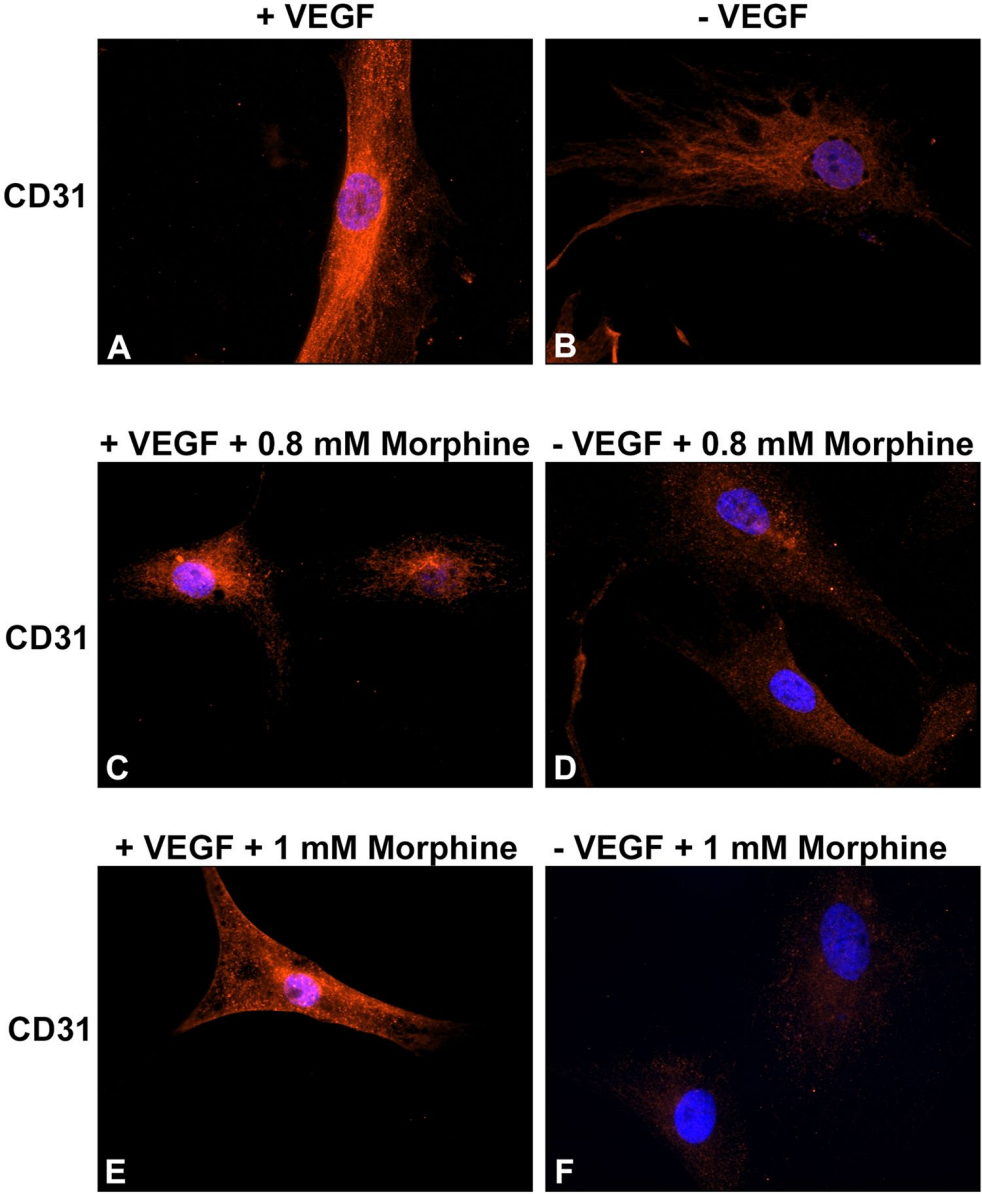
Immunofluorescence images of vMSCs exposed to different concentration of morphine sulphate and immunolabeled for the endothelial CD31 protein. (**A**) vMSCs exposed to VEGF for 7 days reveal a positive staining for CD31 protein marker (red). (**B**) vMSC grown in absence of VEGF for 7 days. A weak signal corresponding to CD31 marker is observed on cellular surface. (**C**) vMSCs exposed to VEGF and 0.8 mM morphine sulphate for 7 days. A reduced red fluorescent signal was observed on cellular surface. (**D**) vMSC grown in absence of VEGF and in presence with 0.8 mM of morphine sulphate for 7 days. Almost no fluorescent signal corresponding to CD31 marker is detected. (E) vMSCs exposed to VEGF and 1 mM morphine sulphate for 7 days. A weak signal is observed on cellular surface. (**F**) vMSC grown in absence of VEGF and in presence with 1 mM of morphine sulphate for 7 days. Almost no fluorescent signal is detected. Images are representative of three independent experiments. All the cells are co-stained with 4,6-diamidino-2-phenylindole (DAPI) (blue) for nuclei visualization. Magnification 600 × for all the images

vMSCs exposed to VEGF for 7 days showed a strong fluorescence signal on their cellular surface corresponding to the endothelial marker CD31 (Fig. 8A). vMSCs exposed to VEGF and 0.8 mM (Fig. 8C) or 1 mM (Fig. 8E) of morphine sulphate showed a strong reduction of the expression of CD31 protein, suggesting an impairing of morphine sulphate on vascular differentiation. Control samples consisting in vMSCs, exposed or not to morphine sulphate, without any VEGF stimulation, showed a weak CD31 expression (Fig. 8B, D, F).

To further confirm the influence of morphine sulphate to impair vascular differentiation on vMSCs, a tubular in vitro assay was carried out on geltrex matrix, exposing cells to VEGF for 7 days.

**Fig. 9:**
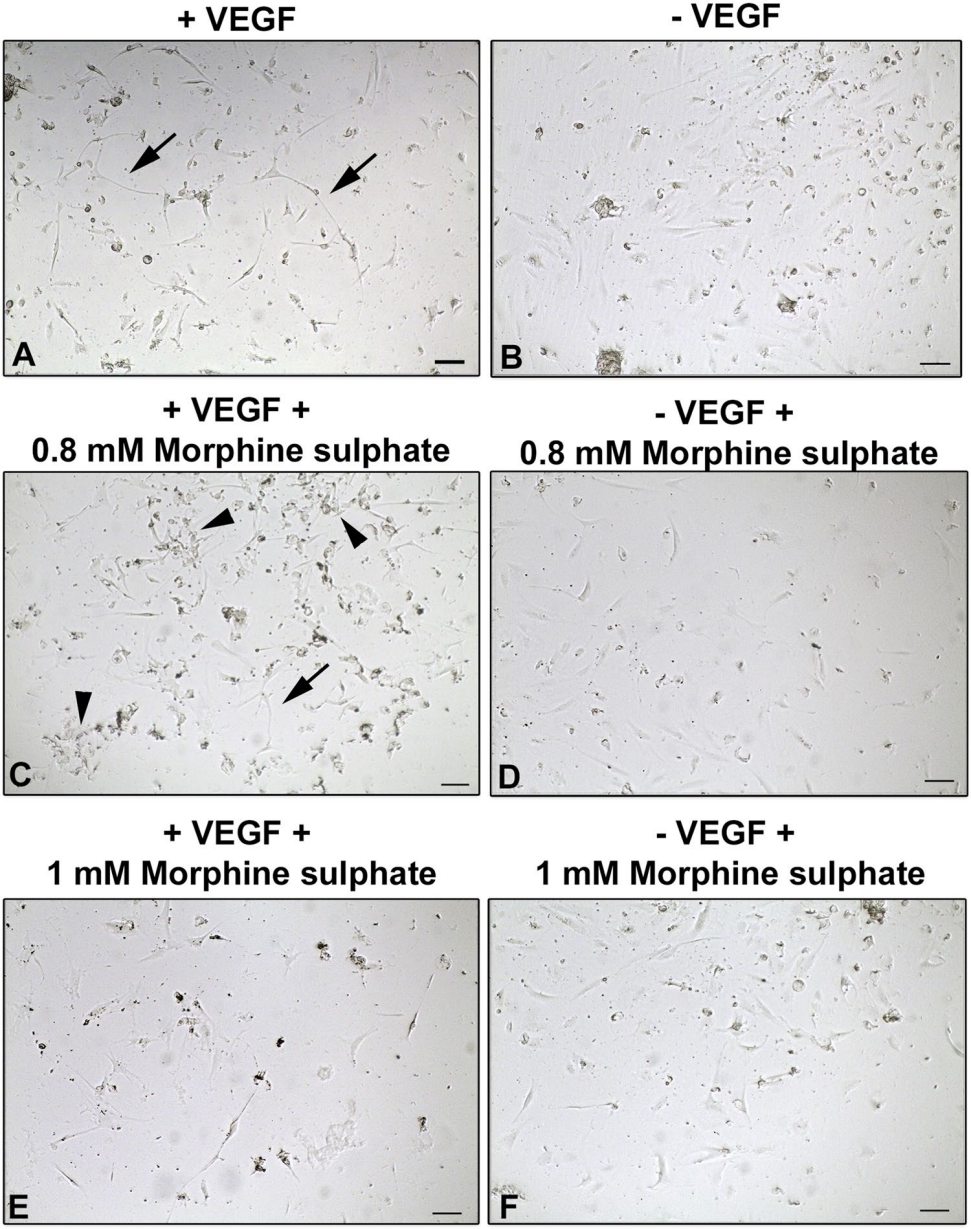
In vitro tubular assay of vMSCs, grown on geltrex matrix and exposed to different concentrations of morphine sulphate and/or VEGF for 7 days. **A** vMSCs exposed to VEGF show a thin and elongated cellular morphology, resembling an endothelial phenotype. Some cells connect each other producing tubular structures (arrows). **B** vMSCs grown in absence of VEGF. Cells showed a fibroblastic and polygonal shape morphology. No tubular structures are detected. **C** vMSCs exposed to VEGF and 0.8 mM morphine. Several cells are dead (arrowheads) while just a few of them show an elongated morphology (arrows). No tubular structures are observed. **D** vMSCs grown in absence of VEGF and in presence with 0.8 mM of morphine sulphate. A few cells are detected showing a fibroblastic and polygonal-like morphology. **E** vMSCs exposed to VEGF and 1 mM morphine. Dead and a few elongated cells are detected. Tubular structures are missing. **F** vMSCs grown in absence of VEGF and in presence with 1 mM of morphine sulphate. A few cells are observed showing a fibroblastic and polygonal-like morphology. Light microscopic images representative of three independent experiments. Bar: 100 μm

Control vMSCs, exposed to VEGF and in absence of morphine sulphate, showed a thin and elongated cellular morphology, resembling an endothelial phenotype (Fig. 9A). Some of these cells were connected to each other, producing a network. Considering vMSCs exposed to both VEGF and morphine sulphate, at concentration of 0.8 mM (Fig. 9C) and 1 mM (Fig. 9E), only few cells displayed an elongated shape (Fig. 9C and E) while most of them remain round shaped, which is a hallmark of cell death (Fig. 9C).

Control cells not exposed to VEGF did not show any morphological modification (Fig. 9B, D, F).

## Discussion

The debate about mechanisms leading to death in cases of fatal opiate intoxications dates more than 50 years and many cherished beliefs have been challenged, including the use of the term “overdose” itself and the role of opiates in polydrug toxicity [12, 13]. Although the nature of opiate-related deaths might vary, it is generally thought to involve the consumption of a certain amount of opioid drug (alone or in combination with other substances) which results in severe respiratory depression, exceeding the physical compensation abilities [3]. In vitro studies have shown that morphine can dose- and time-dependently affect the viability of vascular endothelial cells and increase nitric oxide concentration and ROS production in comparison to control cells [7]. Besides the induction of ROS, aortic endothelium exposed to morphine shows a decrease in the acetylcholine-induced relaxation, which might, in addition to hypoxemia, increase the pulmonary artery pressure throughout the lungs [3, 14].

In such a complex scenario, the purpose of our study was to assess the effect of opiate exposure on vMSCs, which are supposed to be involved in the reparation processes after damages related to the respiratory depression occurring during opiate intoxications.

The effect of incremental doses of morphine sulphate on vMSCs was investigated through an extensive set of techniques demonstrating detrimental consequences on cellular function. In particular, such effects involve a decrease of the cellular metabolic activity at the MTT assay test [15], reflecting a reduction of viability and proliferation associated to cytotoxic effects. Impairment of vMSCs proliferation was confirmed through the BrdU assay, testing bromodeoxyuridine incorporation in DNA of actively proliferating cells. This result also suggested a pro-oxidant and pro-senescence effect related to opiates exposure [11]. On this basis, pro-oxidant effects were confirmed through a carboxy-H2DCFDA assay showing a significant increase in intracellular ROS after morphine exposure as previously reported from in vivo studies carried out in murine models [14].

Apoptotic markers were also evaluated to detect whether cell treatment with morphine might activate the cell death. In our study, despite a dose-dependent increase of pro-caspase 3, the inactive pro-enzyme, only vMSCs exposed to 1 mM of morphine sulphate clearly showed the cleaved fragment of caspase 3, demonstrating that the apoptotic signalling events have occurred.

The pro-senescence effect suggested from BrdU assay results was supported from further tests. A senescence marker, β-galactosidase, was expressed in vMSCs after the exposure to incremental doses of morphine sulphate, demonstrating its pro-senescence effects. β-galactosidase [16, 17] is a lysosomal enzyme involved in autophagy which might be upregulated from ROS production related to morphine exposure. As a further confirmation, both senescent-related markers p21^WAF1/CIP1^ and p16^INK4^ were upregulated at 0.1 mM, 0.4 and 0.8 mM exposure to morphine sulphate.

P21^WAF1/CIP1^ is an inhibitor of apoptosis thus leading to cellular senescence. Interestingly after upregulating following a dose-dependent fashion, at 1 mM of morphine sulphate treatment, a dramatic downregulation of P21^WAF1/CIP1^ to the level of control samples was observed. Actually, this is not surprising considering the consensual activation of caspase 3, which specifically cleaves P21^WAF1/CIP1^ at 1 mM of morphine sulphate treatment [18, 19].

Concentrations of p16^INK4^ increase dramatically as tissue ages, being a biomarker of cellular senescence. Regulation of p16 is complex and involves the interaction of several transcription factors [18–20]. Downregulation at higher morphine sulphate concentration (1 mM) is probably related to the consensual activation of apoptosis signalling. Similarly to p21^WAF1/CIP1^, p16^INK4^ upregulates when the insult to the cell remains tolerable, being downregulated when morphine sulphate exposure exceeds the repair ability of the cell [18–20].

As observed at TEM analysis, effects of morphine sulphate exposure on vMSCs also involve ultrastructural modification, including a round-shaped morphology, which on the contrary is fibroblast-like in control cells; and the development of long-shaped mitochondria associated to a higher number of lysosomes and autophagic vesicles. Finally, the vascular differentiation of vMSCs was impaired when tested both by immunofluorescence and by a tubular in vitro assay on geltrex matrix. Control cells showed a thin, elongated cellular morphology and were partly connected one to each other, producing a network resembling capillary tube formation. However, after the exposure to morphine sulphate, only a few cells showed an elongated shape, whereas the majority remained round shaped, suggesting cell degeneration until death. These further findings are consistent with previous in vivo and in vitro studies, in which matrigel assay showed impaired angiogenesis in animals and reduced tubular formation in cultured endothelial cells treated with morphine [21].

Taken together, although an evident relation between morphine exposition and vMSCs death was not observed, the results of the study showed a clear cell impairment and activation of the apoptotic signalling. Albeit these findings should be confirmed in vivo, they might suggest an impairment of the mechanism of repair and regeneration the endothelium following exposures to morphine, which might particularly play a role in cases of multiple administrations. The impaired tissue regeneration and repair activity, due to the progressive reduction of the functional reserve of vMSCs, might be a contributory factor in the deadly imbalance between opioid-induced respiratory depression and body’s ability to compensate for the drug effect [3, 22]. A reduction in the individual’s baseline endothelium function might have a role in the death of very experienced and tolerant opioid users, which are the typical opiates-overdose victims [12, 22]. Moreover, an overdose fatality usually occurs after a prolonged period of respiratory depression [23, 24]. During this prolonged agony period, tissue regeneration and repair might have time to operate and thus an impairment of these functions could contribute to death.

A limit of this study is that all the tests were performed in vitro: clearly this setting is very different from in vivo conditions and thus results must be interpreted with caution. On the other hand, the in vitro setting could be considered also a key strength of this study. Indeed, opiate overdose is a multi-factorial process; thus, studying the role of a single contributory factor can be very difficult and sometimes misleading on a real in vivo scenario. Further studies focused on the analysis of the same markers on autoptic material (an “ex vivo” study) are needed to confirm these preliminary results.

Although confirmation studies are required, the present study provides new insights into the effects of morphine on the vascular endothelium. The results of this study seem to be encouraging and suggest that the approach based on morphological and immunofluorescence methodologies may have a high potential also to analyze and comprehend the mechanisms of death. In particular, although further studies are mandatory on real fatal opiate intoxications, the application of these techniques in the future may lead to the identification of new markers and morphological parameters useful as complementary investigations for drug-related deaths.

## Data Availability

Data are available on request.
